# Synchronous rectal cancer and extranodal NK/T-cell lymphoma, nasal type: A case report

**DOI:** 10.1097/MD.0000000000049775

**Published:** 2026-07-17

**Authors:** Guoxin Wang, Wenzhen Li, Bingchan Sun, Mingfu Tong

**Affiliations:** a Department of Gastroenterology, Affiliated Hospital of Jiujiang University, Jiujiang, Jiangxi, China; b The First Clinical College of Gannan Medical University, Ganzhou, China; c Department of Gastroenterology, People’s Hospital of Lushan, Jiujiang, Jiangxi, China; d School of Health Science and Engineering, University of Shanghai for Science and Technology, Shanghai, China; e Institute of Digestive Diseases, Jiujiang University, Jiujiang, Jiangxi, China.

**Keywords:** ENKTL-NT, rectal cancer, synchronous primary malignancies, treatment

## Abstract

**Rationale:**

Synchronous primary malignancies involving solid tumors and aggressive lymphomas, such as extranodal NK/T-cell lymphoma, nasal type (ENKTL-NT), are exceedingly rare and pose significant diagnostic and therapeutic challenges. To our knowledge, no previously reported case of synchronous rectal adenocarcinoma and ENKTL-NT has been identified in the available literature.

**Patient concerns:**

A 64-year-old man presented with malaise, loss of appetite, toothache, gingival swelling, and a persistent facial sinus with purulent discharge.

**Diagnoses:**

Colonoscopy and histopathological examination confirmed moderately differentiated rectal adenocarcinoma with liver and sacral bone metastases. Histopathological and immunohistochemical analysis of the facial tissue confirmed ENKTL-NT, which was positive for Epstein‑Barr virus‑encoded small RNA, CD3, CD56, and T‑cell intracellular antigen-1.

**Interventions:**

The patient received 2 cycles of CHOP (cyclophosphamide, hydroxydaunorubicin/doxorubicin, Oncovin/vincristine, and prednisone) chemotherapy before a definitive diagnosis was established. Owing to rapid disease progression and poor nutritional status, no further lymphoma-specific therapy was feasible.

**Outcomes:**

The patient died 3 months after diagnosis, underscoring the aggressive nature of ENKTL-NT and the risks associated with empirical chemotherapy.

**Lessons:**

This case highlights the critical need for early and accurate histopathological diagnosis in suspected lymphoma cases and emphasizes a multidisciplinary strategy to optimize management when synchronous aggressive malignancies are present. Anthracycline-based regimens should be avoided in ENKTL-NT.

## 1. Introduction

Synchronous primary malignancies are defined as 2 or more independent primary malignant tumors diagnosed simultaneously or successively within 6 months in the same patient.^[1]^ Although their incidence is increasing annually with advances in medical technology,^[2]^ the coexistence of solid tumors with highly aggressive lymphomas, such as extranodal NK/T-cell lymphoma, nasal type (ENKTL-NT), remains exceedingly rare and poses significant challenges for clinical diagnosis and treatment.

ENKTL-NT is an aggressive Epstein–Barr virus (EBV)-associated lymphoma that typically originates in the nasal cavity or upper aerodigestive tract.^[3]^ Its diagnosis requires histopathologic and immunohistochemical confirmation, including the expression of CD3, CD56, cytotoxic markers (e.g., T‑cell intracellular antigen-1, granzyme B), and positive Epstein‑Barr virus‑encoded small RNA in situ hybridization.^[4]^ Due to its nonspecific early symptoms, it is often misdiagnosed as chronic infection, leading to delayed treatment.

In this paper, we report a case of synchronous rectal cancer and ENKTL-NT, a combination that, to our knowledge, has not been previously described in the literature. This case highlights the diagnostic complexity and the critical role of multidisciplinary collaboration in managing such rare presentations.

## 2. Case presentation

**Figure 1.**
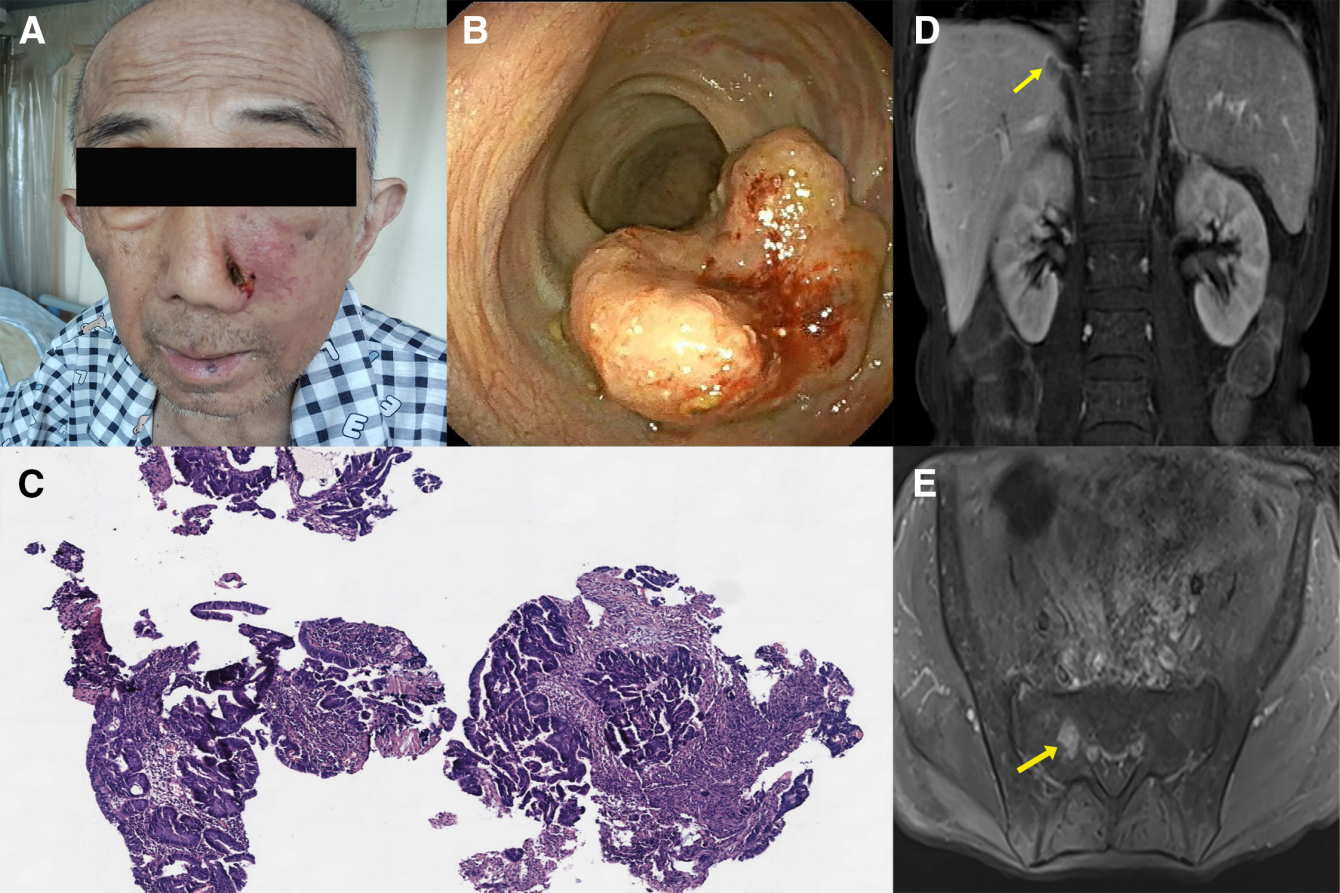
Physical examination findings and diagnostic evaluation of rectal adenocarcinoma. (A) A 0.5-cm sinus tract with purulent discharge on the left nasal ala, (B) colonoscopy revealed a cauliflower-shaped mass in the mid-rectum, (C) rectal biopsy showed moderately differentiated adenocarcinoma with atypical glandular formation (20×), (D) enhanced MRI revealed a metastatic lesion in the right sacrum, (E) enhanced MRI revealed a metastatic lesion in the right sacrum. MRI = magnetic resonance imaging.

A 64-year-old man presented with malaise and loss of appetite for 5 months. In addition, he had toothache and swollen gums and took intermittent antibiotics for toothache and moderate fever. The physical examination was notable for pallor and a sinus tract about 0.5 cm in diameter on the left side of his nasal ala, with visible purulent secretions (Fig. 1A). Laboratory exams showed anemia, low nutrition, a persistent increase in inflammatory indicators, abnormal tumor markers, and positive fecal occult blood.

Colonoscopy revealed a cauliflower-shaped mass in the mid-rectum (Fig. 1B), and a biopsy confirmed moderately differentiated adenocarcinoma (Fig. 1C). Enhanced magnetic resonance imaging showed an abnormal signal shadow in the S7 segment of the liver and right sacrum, which were considered metastatic lesions (Fig. 1D and E).

**Figure 2.**
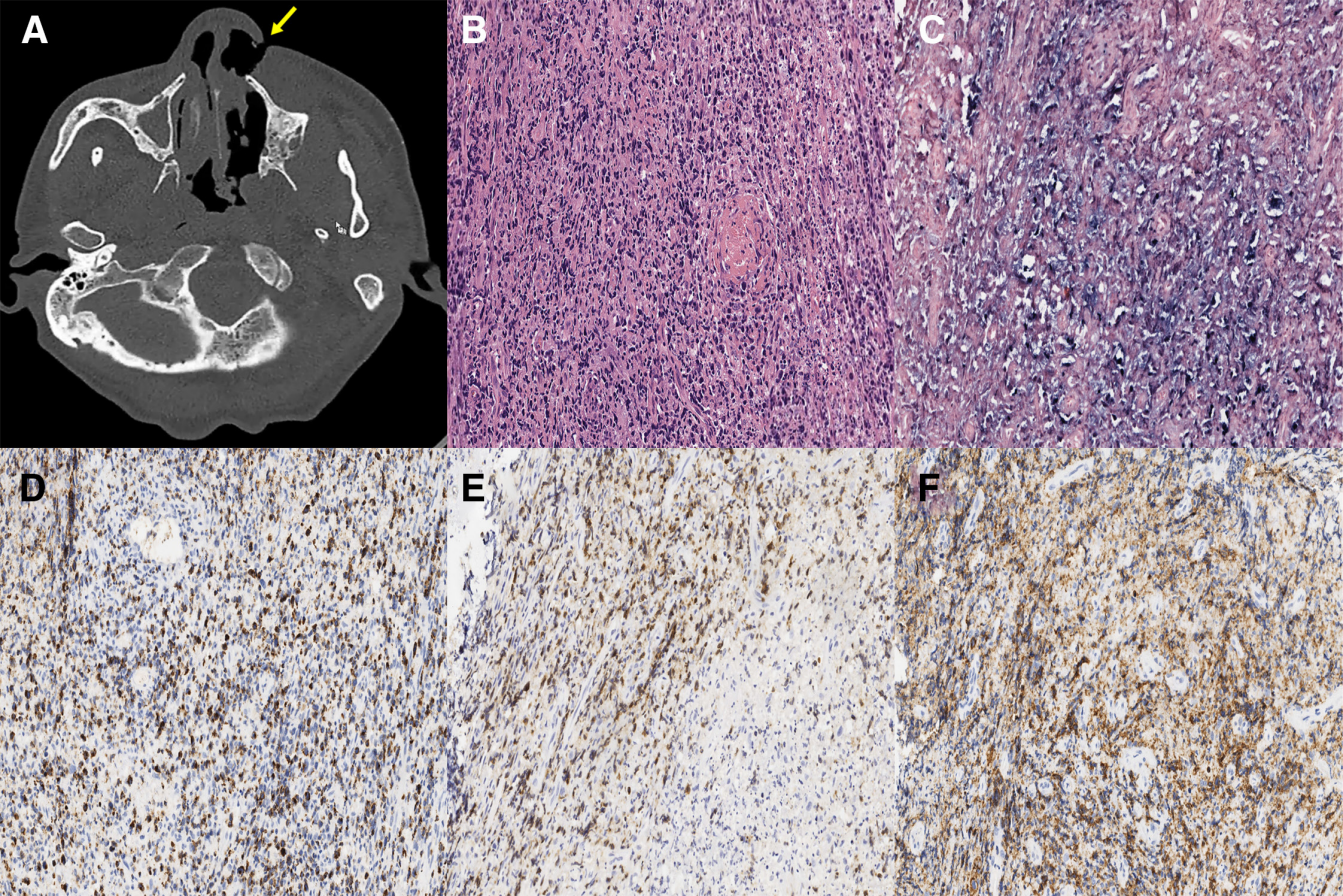
Imaging and pathological findings of extranodal NK/T-cell lymphoma, nasal type. (A) Contrast-enhanced CT showed a mass in the left anterior cranial fossa extending to the left facial region, (B) H&E staining of the nasal mass showed diffuse infiltration of atypical lymphoid cells, (C) EBER in situ hybridization showed diffuse nuclear positivity in tumor cells (100×), (D) tumor cells were positive for TIA-1 (10×), (E) tumor cells were positive for CD3 (10×), (F) tumor cells were positive for CD56 (100×). CT = computed tomography, EBER = Epstein‑Barr virus‑encoded small RNA, NK = natural killer, TIA-1 = T‑cell intracellular antigen-1.

Contrast-enhanced computed tomography of the nose showed a mass in the left anterior cranial fossa with spread to the left side of the face (Fig. 2A). The facial ulcerated tissue was partly resected, and a large number of atypical lymphocytes were observed by hematoxylin-eosin staining (Fig. 2B). Epstein‑Barr virus‑encoded small RNA in situ hybridization showed nuclear positivity in tumor cells, and immunohistochemical analysis demonstrated positivity for T‑cell intracellular antigen-1, CD3, and CD56, confirming the diagnosis of ENKTL-NT (Fig. 2C–F).

The patient was diagnosed with synchronous stage IV rectal adenocarcinoma and ENKTL-NT, having previously received 2 cycles of CHOP (cyclophosphamide, hydroxydaunorubicin/doxorubicin, Oncovin/vincristine, and prednisone) chemotherapy. Following the confirmation of ENKTL-NT, the patient’s rapid disease progression and severely impaired systemic nutritional status precluded any further lymphoma-specific therapy. The patient ultimately succumbed 3 months after diagnosis. The complete clinical timeline of this patient is shown in Figure S1, Supplemental Digital Content 1.

## 3. Discussion

ENKTL-NT is a rare and clinically deceptive disease that often presents with nasal obstruction and abnormal secretions accompanied by systemic symptoms such as fever, leading to frequent misdiagnosis as an infectious condition. When tumor cells invade surrounding tissues, they destroy the midfacial structure.^[5]^ As demonstrated in our case, the patient was initially misdiagnosed with a bacterial infection due to fever and facial ulceration, leading to frequent antibiotic use and delayed lymphoma diagnosis.

Despite numerous case reports having revealed the clinical characteristics of ENKTL-NT, with its rapid progression and poor prognosis, the absence of an optimal treatment regimen remains a challenging issue in clinical practice.^[6–8]^ Unlike treatment regimens for non-Hodgkin lymphoma, ENKTL-NT tumors exhibit high expression of multidrug resistance proteins, rendering anthracycline-based therapies such as CHOP generally ineffective and potentially accelerating disease progression.^[9]^ However, before a definitive diagnosis was established, the patient had received 2 cycles of empirical CHOP chemotherapy. Given the poor responsiveness of ENKTL-NT to anthracycline-based regimens, CHOP was unlikely to achieve effective disease control and may have delayed the initiation of appropriate lymphoma-directed therapy. This underscores the risks associated with initiating empirical chemotherapy in the absence of a definitive diagnosis and highlights the necessity of prompt pathological confirmation in suspected ENKTL-NT.

The current standard of care is a stratified treatment strategy based on non-anthracycline-containing regimens. For early-stage patients, regimens incorporating L-asparaginase (L-aspa) (e.g., SMILE, P-GemOX) combined with high-dose radiotherapy (≥50 Gy) form the cornerstone of curative treatment, with sequential chemoradiotherapy being widely adopted due to its superior tolerability. For advanced, relapsed, or refractory patients, intensive chemotherapy centered on L-aspa (e.g., SMILE, DDGP) is pivotal for inducing remission. Following remission, autologous or allogeneic hematopoietic stem cell transplantation is recommended to improve survival outcomes.^[10,11]^ In recent years, immune checkpoint inhibitors targeting PD-1 have demonstrated remarkable efficacy in patients with recurrent or refractory disease, offering new hope for this patient population.^[12]^ ENKTL-NT exhibits a strong association with EBV infection, with plasma EBV DNA levels frequently correlating with symptom severity.^[13]^ Positron emission tomography/computed tomography (PET/CT) proves valuable for staging and detecting extra-nasal lesions.^[14]^ Consequently, plasma EBV DNA and PET/CT have become essential tools for assessing tumor burden, dynamically predicting prognosis, and guiding treatment decisions.^[15,16]^ In the present case, PET/CT and plasma EBV DNA quantification were not performed owing to the patient’s rapid clinical deterioration and limitations in local medical resources. This represents a limitation of the clinical assessment.

Notably, current European Society for Medical Oncology guidelines for metastatic colorectal cancer emphasize individualized treatment decision-making based on disease burden, treatment goals, performance status, organ function, comorbidities, and patient-related factors.^[17]^ In this case, colorectal cancer-specific therapy was not initiated because the patient’s clinical course was dominated by aggressive ENKTL-NT, prior CHOP exposure, poor general condition, anemia, malnutrition, and persistent inflammatory status, which limited the feasibility of further systemic antitumor treatment.

To our knowledge, no previously reported case of synchronous rectal adenocarcinoma and ENKTL-NT has been described in the literature. The clinical management of synchronous malignancies remains a significant challenge in oncology, particularly when dealing with histologically distinct and highly aggressive diseases, due to the absence of standardized guidelines for their unified management. For such complex cases, multidisciplinary collaboration is required to prioritize intervention for highly aggressive malignancies while developing appropriate treatment strategies for other tumors.^[18,19]^

## 4. Conclusion

This case report presents a documented instance of coexisting rectal adenocarcinoma and ENKTL-NT, highlighting the significant diagnostic and therapeutic challenges posed by concurrent dual primary malignancies. Clinical practice indicates that such scenarios necessitate a multidisciplinary approach to management, where intervention targets the more aggressive tumor while concurrently addressing the other neoplasm, thereby enabling the formulation of individualized, stratified treatment strategies for the patient.

## Acknowledgments

This work was supported by the General Program of Natural Science Foundation of Jiangxi Province (No. 20224BAB206110) and Science and Technology Program of Traditional Chinese Medicine of Jiangxi Provincial (No. 2023A0338). The authors acknowledge the use of BioRender.com for creating Figure S1, Supplemental Digital Content 1 (Biorender.com).

## Author contributions

**Conceptualization:** Mingfu Tong.

**Resources:** Wenzhen Li.

**Data curation:** Bingchan Sun.

**Visualization:** Bingchan Sun.

**Funding acquisition:** Mingfu Tong.

**Supervision:** Mingfu Tong.

**Writing – original draft:** Guoxin Wang.

**Writing – review & editing:** Guoxin Wang, Wenzhen Li, Mingfu Tong.



## References

[1] Ye X Liu X Yin N. Successful first-line treatment of simultaneous multiple primary malignancies of lung adenocarcinoma and renal clear cell carcinoma: a case report. Front Immunol. 2022;13:956519. 35979370 10.3389/fimmu.2022.956519 PMC9376962

[2] Zheng Y Sun Y Kuai Y. Gene expression profiling for the diagnosis of multiple primary malignant tumors. Cancer Cell Int. 2021;21:47. 33514366 10.1186/s12935-021-01748-8 PMC7846996

[3] Sanchez-Romero C Bologna-Molina R Paes De Almeida O. Extranodal NK/T cell lymphoma, nasal type: an updated overview. Crit Rev Oncol Hematol. 2021;159:103237. 33493634 10.1016/j.critrevonc.2021.103237

[4] Tse E Kwong Y. Diagnosis and management of extranodal NK/T cell lymphoma nasal type. Expert Rev Hematol. 2016;9:861–71. 27347812 10.1080/17474086.2016.1206465

[5] Takahara M Kumai T Kishibe K Nagato T Harabuchi Y. Extranodal NK/T-cell lymphoma, nasal type: genetic, biologic, and clinical aspects with a central focus on Epstein-Barr virus relation. Microorganisms. 2021;9:1381. 34202088 10.3390/microorganisms9071381 PMC8304202

[6] Davila YNC Leon VHP Astudillo ST Erazo GML Rubio JDG. Extranodal NK/T-cell lymphoma, nasal type, extranasal and ulcerative blister variant, case report. Ann Dermatol. 2023;35:S304–9. 38061727 10.5021/ad.21.317 PMC10727907

[7] Mao S Diao C Cao L. Primary small intestinal extranodal NK/T cell lymphoma, nasal type with kidney involvement: a rare case report and literature review. Diagn Pathol. 2022;17:75. 36199094 10.1186/s13000-022-01254-z PMC9533626

[8] Han Y Wei S Zhang Y Wang L Xu X. Extranodal nasal-type NK/T-cell lymphoma with CD20 positive: a case report and review of literature. Indian J Pathol Microbiol. 2024;67:459–62. 38391360 10.4103/ijpm.ijpm_32_22

[9] Tse E Kwong Y. The diagnosis and management of NK/T-cell lymphomas. J Hematol Oncol. 2017;10:85. 28410601 10.1186/s13045-017-0452-9 PMC5391564

[10] Allen PB Lechowicz MJ. Management of NK/T-cell lymphoma, nasal type. J Oncol Pract. 2019;15:513–20. 31600461 10.1200/JOP.18.00719 PMC6790879

[11] Tse E Zhao W Xiong J Kwong Y. How we treat NK/T-cell lymphomas. J Hematol Oncol. 2022;15:74. 35659326 10.1186/s13045-022-01293-5 PMC9164389

[12] Kim SJ Lim JQ Yoon SE. Efficacy of combined CD38 and PD-1 inhibition with isatuximab and cemiplimab for relapsed/refractory NK/T-cell lymphoma. Blood. 2025;146:155–66. 40073374 10.1182/blood.2024027109

[13] Zhang YT Li N Wang JJ Li YM Huang H Zhang YJ. Association of B symptoms with plasma EBV-DNA copy number and cytokine profiles in patients with nasal type Extranodal Natural Killer/T-Cell Lymphoma (ENKTL): a mechanism and prognostic study. Int J Radiation Oncol Biol Phys. 2021;111:e304.

[14] Su H Su M Jiang M Zou L Jiang C Tian R. [18 F]FDG PET/CT is superior to [68 Ga]Ga-FAPI PET/CT for lesion detection in extranodal NK/T-cell lymphoma, nasal type. Clin Nucl Med. 2025;50:992–7. 40730507 10.1097/RLU.0000000000006082

[15] Xu P Guo R You J. Dynamic evaluation of the prognostic value of 18F-FDG PET/CT in extranodal NK/T-cell lymphoma, nasal type. Ann Hematol. 2021;100:1039–47. 33634350 10.1007/s00277-021-04466-3

[16] Suzuki R Yamaguchi M Izutsu K. Prospective measurement of Epstein-Barr virus-DNA in plasma and peripheral blood mononuclear cells of extranodal NK/T-cell lymphoma, nasal type. Blood. 2011;118:6018–22. 21984805 10.1182/blood-2011-05-354142

[17] Cremolini C Chalabi M Elez E. Metastatic colorectal cancer: ESMO clinical practice guideline for diagnosis, treatment and follow-up. Ann Oncol. 2026;37:759–76. 41990853 10.1016/j.annonc.2026.03.005

[18] Al-Gahmi A Alhuthali M Alrehaili M Baltow B Tashkandi E. Unusual synchronous association of solid tumors with hematological malignancies in multiple primary cancers: case series and literature review. Case Rep Oncol. 2021;14:352–64. 33776729 10.1159/000514147 PMC7983565

[19] Dayer N Fasquelle F Salati E Dietrich G. Multiple primary malignancies: synchronous lymphoma, pancreatic neuroendocrine tumour and colorectal cancer. BMJ Case Rep. 2021;14:e241938. 10.1136/bcr-2021-241938 PMC8183193 34088689

