# Supplementary figures and images for: Synchronous rectal cancer and extranodal NK/T-cell lymphoma, nasal type: A case report
Source: Medicine (Baltimore). 2026 Jul 17;105(29):e49775. doi: 10.1097/MD.0000000000049775 (PMC13384672; doi:10.1097/MD.0000000000049775)

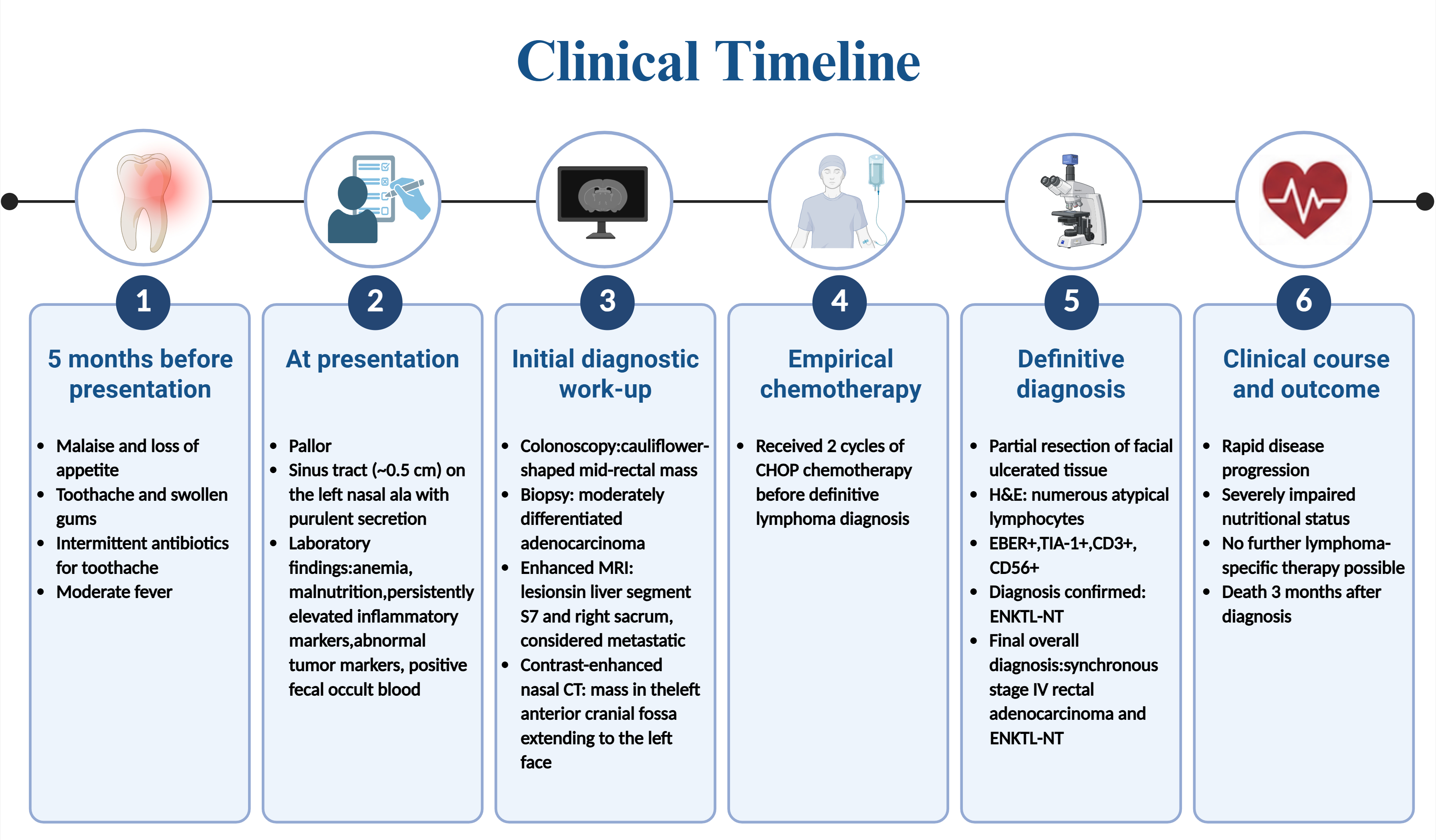

Supplement: Supplementary file 1 [file medi-105-e49775-s001.tif]
